# A change is needed in the landscape of preclinical models to test drugs that target aging

**DOI:** 10.31491/apt.2024.12.162

**Published:** 2024-12-28

**Authors:** Warren Ladiges

**Affiliations:** a Department of Comparative Medicine, School of Medicine, University of Washington, Seattle, WA, USA

**Keywords:** Aging intervention, drugs and drug combinations, preclinical animal models, house crickets, aging mice, translational drug testing

## Abstract

Efficient and reproducible preclinical models for testing drugs or drug combinations that target aging are vital to develop a pipeline that results in a predictable outcome for geroscience research and geriatric medicine. Lifespan as a readout test in laboratory mice has been successful in identifying several drugs that robustly enhance healthy aging, and has provided impactful information for moving to clinical studies. However, it is a costly and time consuming process (about three years), and poorly designed to test drug combinations. Therefore, a more efficient pipeline is needed that would provide an increased number of drugs or drug combinations with promising and predictable outcomes for first in human studies in a shorter time frame. This editorial discusses an alternate system involving prescreening in an invertebrate model (the domestic house cricket) followed by short term cross sectional testing in aging mice. The time frame is about six months, and the system is simple enough to allow testing of multiple drugs concurrently. The cricket to mouse pipeline provides a logical and preclinical translational approach to identify drugs that have the potential to enhance human health at later ages of life.

Testing drugs that target aging is becoming an area of high interest in geroscience research and geriatric medicine. Efficient and reproducible preclinical models are vital to develop a pipeline that results in a predictable outcome for testing a drug or drug combinations in older people. Traditionally, lifespan in laboratory mice has been used as a readout to provide much of the rationale to consider additional aging studies. This approach has identified several drugs with a high impact on enhancing healthy aging. However, there are limitations to screening large numbers of drugs or drug combinations because of the extensive mouse numbers required and the extended time (around 3 years) to conduct lifespan studies. In addition, mouse lifespan studies are not designed to efficiently test drug combinations, which can consist of multiple drugs as a cocktail [1]. These limitations have resulted in a relatively low number of drugs shown to actually increase lifespan, indirectly contributing to the lack of translational success. Changes are needed to develop a more efficient pipeline that would provide an increased number of drugs or drug combinations with promising and predictable outcomes for first in human studies in a much shorter time frame. This editorial discusses an alternate system that could be developed into a rapid and efficient pipeline that would screen drugs with a high outcome predictability. The system involves prescreening in an invertebrate model followed by short term cross sectional testing in aging mice. The time frame for a single drug is about six months, but the system is simple enough to allow testing of multiple drugs concurrently.

The invertebrate model of choice is not a nematode or drosophila (flies), but instead the domestic house cricket (*Acheta domesticus*). There are many advantages of this insect species as an aging model for this purpose [2]. First, they have a relatively short testable lifespan of about two months. Second, they are omnivores and can eat the same foods as humans, and drugs can easily be delivered in the food. Third, heterogenous outbred crickets can readily be obtained from commercial sources (for example, Fluker Farms, Port Allen, LA, USA), at a very inexpensive rate, or in-house breeding can easily be set up. Fourth, no animal use protocol is necessary so drug testing experiments are not delayed waiting for approval. Fifth, crickets are very amenable to behavioral and frailty assessments such as the Y maze, open field with EZ tracking software, escape paradigm, and treadmill running, thus providing relevant phenotypic data. Sixth, crickets have simple but surprisingly similar anatomic and histological systems compared to mammals. There is extensive evidence of the development of age-related lesions (geropathology), which can be quantitatively graded according to the degree of severity with increasing age and used as an additional readout to help determine the effect of a drug on aging (unpublished observations). Seventh, crickets, like other invertebrates, have conserved pathways of aging so immunohistochemistry and numerous other bench assessments can be performed as needed. Eighth, both sexes can be tested since males and females are easily recognized by external body features.

Drugs can be tested in crickets using a two-pronged approach. The first is a lifespan trial (after establishing the drug dose) that takes about 8 weeks. This is feasible as we have shown that drugs, such as rapamycin, acarbose, and phenylbutyrate with validated anti-aging effects in mammalian models, robustly extend lifespan in crickets (unpublished observations). Drugs that extend cricket lifespan are qualified to move to the second prescreening approach involving a cricket cross sectional trial, which takes about 6 weeks, and ends with phenotypic assessments and organ geropathology. Drugs or drug combinations that significantly enhance resilience to aging in the cross sectional cricket test qualify for testing in the mammalian phase, a cross-sectional approach using middle-aged mice. This phase can be accomplished in about 3 months provided that mice at this age are readily available. In the United States, the National Institute on Aging subsidizes an Aged Rodent Colony providing convenient access to aging mice. Mouse strains that are typically available are C57BL/6 and CB6F1 hybrid cross. Drugs are added to standard rodent chow and fed to middle-aged mice (20 months of age) for the 3-month period ending with phenotypic assessments and quantitative geropathology, immunohistochemistry, and other molecular assessments as needed [3].

Drugs or drug combinations that perform significantly better compared to placebo would then be ideal candidates for a variety of additional testing considerations focused on preparations and qualifications for clinical studies. Ideally some type of *in vitro* human cell or tissue culture system, such as stem cell driven organoids, tissue on a chip or organ slice cultures, would provide additional translational relevance. However, the technology for these *in vitro* human systems is not yet fully developed for testing drugs that target aging.

A final consideration is whether drug prescreening in aging crickets would at some point provide enough translational information to bypass mice and test directly in a to be developed *in vitro* human tissue culture system. This is a relevant question that would have a huge impact on the way drugs could rapidly and reliably be tested in the preclinical phase to target aging and subsequently be considered for clinical trials. Once the technology for a reproducible *in vitro* human tissue culture system is established this type of pipeline could very well be a mainstay in qualifying drugs for clinical studies in aging individuals. Currently, the cricket to mouse pipeline provides a logical and preclinical translational approach to identify drugs that have the potential to enhance human health at later ages of life.
